# Systemic lupus erythematosus, antiphospholipid syndrome and Hashimoto thyroiditis occurring in a patient with Niemann-Pick disease: a second case

**DOI:** 10.11604/pamj.2020.36.367.25116

**Published:** 2020-08-28

**Authors:** Wafa Baya, Fatma Ben Fredj, Imen Ben Hassine, Jihed Anoun, Anis Mzabi, Monia Karmani, Amel Rezgui, Mohamed Adnane Laatiri, Chedia Laouani Kechrid

**Affiliations:** 1 Université de Sousse, Faculté de Médecine de Sousse, Hôpital Sahloul, Service de Médecine Interne, Unité de Gérontologie et Gériatrie Clinique, 4000, Sousse, Tunisie,; 2 Service d´Hématologie Clinique, Hôpital Universitaire Fattouma Bourguiba, Faculté de Médecine, Université de Monastir, 5019, Monastir, Tunisie

**Keywords:** Niemann-Pick disease, systemic lupus erythematous, antiphospholipid syndrome, Hashimoto thyroiditis

## Abstract

Lysosomial diseases and autoimmune diseases are systemic disorders. Their clinical manifestations can overlap with the broad spectrum of one another. Their association has been rarely reported. We report a new case of systemic lupus erythematous (SLE) associated to antiphospholipid syndrome (APS) and Hashimoto thyroiditis occurring in Niemann-Pick disease (NPD) type B patient. A 42-year-old woman with a familial history of NPD was diagnosed with a NPD type B at the age of ten. Twenty years later (2008), she complained of inflammatory arthralgia with acute dyspnea. She was diagnosed with SLE (according to ACR criteria) and Hashimoto disease with positive IgG anti-cardiolipin and IgA anti-beta2 glycoprotein. In 2018, she presented a left segmental pulmonary embolism. Antiphospholipid syndrome was retained. She was treated with steroids, hydroxychloroquine, anticoagulation therapy and levothyroxine. Her SLE treatment was re-enforced by cyclophosphamide because of corticosteroid dependence and recurrent hemolytic crises.

## Introduction

Lysosomial diseases and autoimmune diseases are systemic disorders. Their clinical manifestations can overlap with the broad spectrum of one another. Their association has been rarely reported. The pathological mechanisms of autoantibodies development within lysosomial disorders are still unknown. High incidence of autoantibodies in Fabry disease [1] and Gaucher disease [2] patients has been reported. Their coexistence with systemic lupus erythematous (SLE) has been reported in few cases. Nevertheless, the association between Niemann-Pick disease (NPD) and SLE has been reported only once [3]. Herein, we report a new case of SLE associated to antiphospholipid syndrome (APS) and Hashimoto thyroiditis occurring in NPD type B patient.

## Patient and observation

A 42-year-old white Tunisian woman with a familial history of NPD was diagnosed with a NPD type B at the age of ten, presenting hepatosplenomegaly (HSM) with anemia. A bone marrow biopsy confirmed the diagnosis then. Folate supplementation was administrated. Twenty years later (2008), she complained of inflammatory arthralgia with acute dyspnea. Physical examination found a persistent HSM and crackles at lung auscultation. Laboratory investigations revealed severe anemia (hemoglobin at 6 g/dl) related to active hemolysis, lymphopenia (700/μl), thrombocytopenia (115000/μl) and IgG positive direct Coombs test. C3 level was low (0.3 g/l). C4 level was normal. Antinuclear antibodies (ANA) were positive in the range of 1: 1600 with positive anti-nucleosome antibodies (ANA). IgG anti-cardiolipin and IgA anti-beta 2 glycoprotein antibodies were positive and their positivity was persistent after 12 weeks. Furthermore, we found a highly increased TSH level with positive anti-thyroid peroxidase antibodies. She was diagnosed with SLE (according to ACR criteria) and Hashimoto disease. She was treated with steroids, Hydroxychloroquine, Aspirin and Levothyroxine. In 2018, she presented a new episode of acute dyspnea. Blood gas showed respiratory alkalosis, hypoxia and hypocapnia. Chest high-resolution computed tomography imaging showed diffuse infiltrative lung disease, extra medullary hematopoiesis and left segmental pulmonary embolism. Antiphospholipid syndrome was retained. Anticoagulant therapy was initiated. Her SLE treatment was re-enforced by cyclophosphamide because of corticosteroid dependence and recurrent hemolytic crises.

## Discussion

Primary sphingomyelinases are autosomal recessive lysosomal lipid storage disorders consequent to mutations in the sphingomyelin phosphodiesterase 1 (SMPD1) gene. These mutations lead to acid sphingomyelinase deficiency [4]. Two types A and B have been categorized, depending on the degree of neurologic system involvement [5]. An intermediate form has also been described [5, 6]. The age onset is variable. It ranges from infancy to adulthood with a progressive evolution. NPD is a systemic disease. Clinical manifestations result from sphingomyelin and other lipids accumulation in hepatocytes and the macrophage-monocytes system. Therefore, multiple organs can be affected. NDP type B is a chronic visceral form with no neuropathic manifestations. Hepatosplenomegaly with a significant splenic volume is the main symptom. Life expectancy is reduced due to respiratory and liver complications. Many differential diagnoses must be kept in mind giving the symptoms diversity including autoimmune disease. Nevertheless, in our case we concluded to the association of NDP to autoimmune diseases.

In fact, adding to systemic manifestations, autoantibodies such as ANA can be positive. This positivity can be non-specific and non-pathogenic in NDP [7] as well as other lysosomial diseases [1, 2]. Still, Murgia *et al*. reported the first case of a 30-year-old woman affected by NPD intertwined with clinical and serological features of SLE [3]. Development of autoantibodies is still not totally understood. Autophagic dysfunction seems to be resulting in an inappropriate response of the immune system like described in SLE. [8] In lysosomial storage disorders such as NPD, undergraded molecules are accumulated and lysosomial enzymes are dysfunctional which lead to autophagic dysregulation. This process results in a chronic stimulus activating the immune system. This leads finally to tissue injury consequent to an inappropriate production of antibodies and immune complexes. The coexistence of autoimmune diseases, whether systemic or organ-specific diseases, is common. SLE and APS are frequently associated. Antiphospholipid antibodies are found positive in 40% of SLE patients [9]. Nevertheless, these two diseases are distinct autoimmune entities. In another hand, SLE associated thyroiditis has been reported up to 51% of the cases [10]. These findings indicate associated-autoimmune-disease systematic screening in SLE patients.

## Conclusion

Clinical and serological SLE features can be entangled with NPD. To our knowledge, and after literature review, this case is the second one highlighting the rare association of NPD and SLE. Furthermore, no cases exposing the coexisting of NPD with antiphospholipid syndrome and/or Hashimoto thyroiditis have been published yet.
